# Bone Fracture in Rett Syndrome: Mechanisms and Prevention Strategies

**DOI:** 10.3390/children10121861

**Published:** 2023-11-27

**Authors:** Carla Caffarelli, Antonella Al Refaie, Caterina Mondillo, Michela De Vita, Leonardo Baldassini, Giuseppe Valacchi, Stefano Gonnelli

**Affiliations:** 1 Department of Medicine, Surgery and Neuroscience, University of Siena, 53100 Siena, Italy; antonella.alrefaie@student.unisi.it (A.A.R.); c.mondillo@student.unisi.it (C.M.); devita13@student.unisi.it (M.D.V.); leo.balda90@libero.it (L.B.); gonnelli@unisi.it (S.G.); 2 Department of Biomedical and Specialist Surgical Sciences, University of Ferrara, 44121 Ferrara, Italy; vlcgpp@unife.it; 3 Animal Science Department, Plants for Human Health Institute, North Carolina Research Campus, North Carolina State University, Kannapolis, NC 27695, USA

**Keywords:** Rett syndrome, fractures, osteoporosis, calcium, vitamin D, bisphosphonates

## Abstract

The present study aimed to evaluate the burden and management of fragility fractures in subjects with Rett syndrome. We searched all relevant medical literature from 1 January 1986 to 30 June 2023 for studies under the search term “Rett syndrome and fracture”. The fracture frequency ranges from a minimum of 13.9% to a maximum of 36.1%. The majority of such fractures occur in lower limb bones and are associated with low bone mineral density. Anticonvulsant use, joint contractures, immobilization, low physical activity, poor nutrition, the genotype, and lower calcium and vitamin D intakes all significantly impair skeletal maturation and bone mass accrual in Rett syndrome patients, making them more susceptible to fragility fractures. This review summarizes the knowledge on risk factors for fragility fracture in patients with Rett syndrome and suggests a possible diagnostic and therapeutic care pathway for improving low bone mineral density and reducing the risk of fragility fractures. The optimization of physical activity, along with adequate nutrition and the intake of calcium and vitamin D supplements, should be recommended. In addition, subjects with Rett syndrome and a history of fracture should consider using bisphosphonates.

## 1. Introduction

Rett syndrome is a rare neurological disorder caused by mutations in the X-linked gene methyl-CpG-binding protein 2 (MECP2), a ubiquitously expressed transcriptional regulator. Characteristic symptoms of patients with Rett syndrome include repetitive hand movements, a loss of acquired speech and motor skills, breathing irregularities, and frequent seizures. Patients with Rett syndrome may also suffer from sporadic episodes of gastrointestinal problems, bruxism, and screaming spells [1,2,3,4]. Bone complications, mostly scoliosis but also osteoporosis and a high rate of fractures, are among the most prevalent non-neurological comorbidities [5]. Several investigations on both humans and animals have found that abnormal osteoblast activity causes a decreased rate of bone formation as opposed to an increase in bone resorption [6]. These studies support the idea that the MECP2 mutation could be associated with the altered epigenetic regulation of bone-related factors and signaling pathways, including the SFRP4/WNT/β-catenin axis and the RANKL/RANK/OPG system [6]. As a result, people with Rett syndrome frequently have a lower bone mass and density than females in the general population. As a result, the fracture rate is almost four times higher than that of the whole population. Fractures frequently occur in the long bones of the arms and legs and can occur on their own as a result of minor trauma or a fall. Rett syndrome also involves spinal bone fractures, which are typically linked to osteoporosis [7,8]. Furthermore, because some Rett syndrome patients have decreased sensitivity to pain and have difficulty communicating any discomfort, it can be challenging to diagnose fractures in these patients.

It is known that bone fragility fractures are the main complications of osteoporosis in adults, and they represent a similar problem in children and adolescents [9]. Secondary osteoporosis is being increasingly recognized among children with chronic conditions and may be caused by the underlying disease or its treatments [9]. A reduced bone mass and bone mineral density, a low calcium intake, a high or low body mass index (BMI), prolonged immobilization and movement impairment, the consumption of carbonated beverages, and the use of corticosteroids or anti-epileptic drugs (AEDs) have been variably associated with fractures in children [9,10]. There is a lack of systematic data on the incidence/prevalence of bone fragility fractures in the literature, despite the fact that multiple studies have indicated the presence of these fractures in subjects with Rett syndrome [7,8]. Similarly, the most important risk factors responsible for bone fragility and fractures in girls with Rett syndrome have not yet been defined [7,8].

This review aimed to: (1) summarize the available data on fragility fracture in patients with Rett syndrome in order to allow for a better understanding of the pathophysiology of bone fragility and (2) suggest a possible diagnostic and therapeutic care pathway for improving low BMD and reducing the fragility fracture risk.

## 2. Materials and Methods

**Figure 1:**
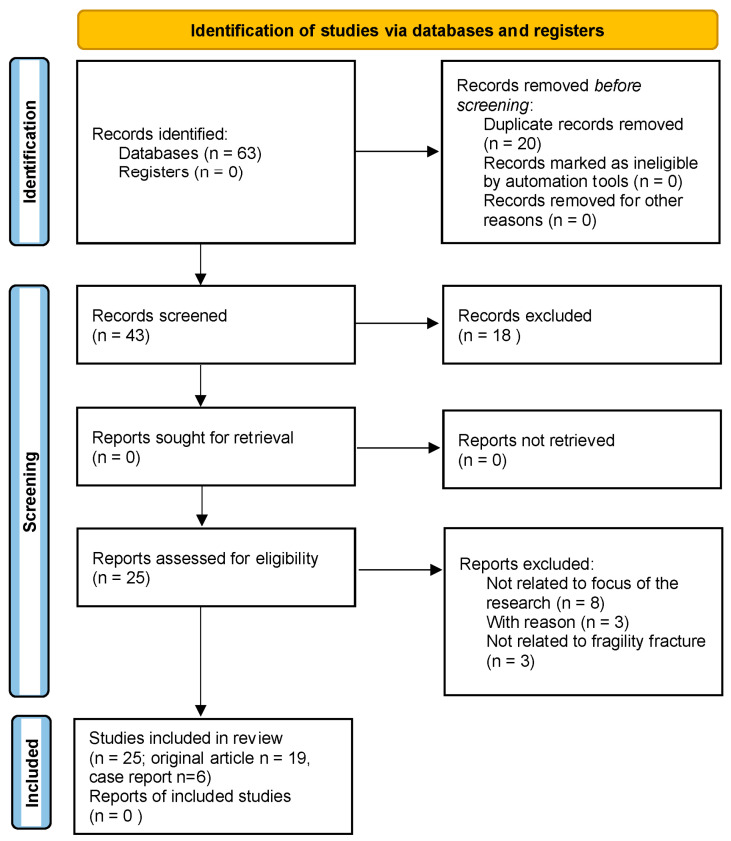
Flow chart of the studies identified and included in the review.

We searched the MEDLINE/PUBMED, Cochrane Library, ClinicalTrials.gov, and SCOPUS databases from 1 January 1986 to 30 June 2023 to identify all relevant English-language medical literature for studies under the search text term: “Rett syndrome AND fractures”. Across these databases, these search terms produced 43 results. From these results, we excluded studies not related to fragility fractures and duplicates. The titles, abstracts and complete texts were screened separately by two authors (AA and CC). All authors had a discussion to resolve any differences. All required data were extracted from the completed studies using a data charting form that was created. The data were extracted and compiled by two authors working together. The data contained details about the study, including its purpose, design, sample, year, and country. The process of selecting the studies for review in adherence with the PRISMA 2020 process is shown in Figure 1.

## 3. Fragility Fractures in Patients with Rett Syndrome

Studies that evaluate the presence of fragility fractures in patients with Rett syndrome are limited, and many are small-group or case reports. The selected studies evaluating fractures in patients with Rett syndrome are summarized in Table 1.

We took into account 19 studies, of which 16 were cross-sectional studies and only 3 were longitudinal studies. The cross-sectional studies we analyzed showed a fracture frequency ranging from a minimum of 13.9% [11] to a maximum of 36.1% [12]. On the other hand, in the few longitudinal studies, the percentage of fractures occurring during the follow-up is 5% [15,22,26]. The presence of fragility fractures in girls with Rett syndrome was first described by Loder RT in 1989 [11]. The most frequent sites where fractures occur are the upper and lower limbs [7,11,12,15]. In particular, from the detailed analysis of the aforementioned studies, it is clear that most fractures affect the lower limbs, especially at the level of the femoral site [7,12]. Namely, the study by Leonard et al. reported that in a group of 100 subjects with Rett syndrome, 34.7% had a history of fragility fracture by the age of 15 years, and in particular, more than one-third of these fractures were located at the femoral site [12]. Moreover, several studies report that the main risk factor for hip fractures in patients with Rett syndrome is immobility [18,26]. It is known that the loss of load is responsible for the loss of bone tissue strain, reductions in bone mass, and, in some cases, disuse osteoporosis. Numerous studies have shown an association between the presence of fractures and decreased BMD values [11,12,14,17,21,24,27,28]. In particular, the study by Jefferson et al. reported that BMD values were particularly low at the femoral neck, the most common site of fracture, with respect to BMD values at the lumbar spine [17]. Only one study showed that patients with Rett syndrome and fractures present a reduction in cortical thickness and in the cortical area [12]. Moreover, the study by Roende et al. showed that the fracture risk in patients with Rett syndrome seems to be associated not only with the reduction in bone mineral density but also with the reduction in bone size [18]. Several studies have reported that the use of AEDs in patients with Rett syndrome increases the risk of fragility fractures [7,16,20]. In particular, phenytoin, phenobarbital, and carbamazepine—AEDs that induce cytochrome P450—are linked to a low bone mass and vitamin D deficiency, which raises the risk of fracture. Moreover, the reduced serum levels of vitamin D that we find in patients with Rett syndrome are also due to decreased sunlight exposure because of an indoor lifestyle but, above all, due to nutritional problems. In particular, there is evidence that patients with Rett syndrome are more prone to feeding difficulties and gastro-intestinal problems. All these factors are believed to contribute to the poorer nutritional status of girls with RTT [16,26]. Therefore, malnutrition can lead to weakened bones and reduced muscle mass, which increases the risk of fragility fractures in patients with Rett syndrome. Regarding the influence of genetic mutations on the risk of fracture, few studies have assessed this aspect [7,20,27]. In particular, Caffarelli et al. reported that the number of patients with fragility fractures prevailed in the group with the most severe mutations (i.e., R270X, R168X, R255X, and R106T) compared to subjects with less severe mutations (i.e., T158 M, R133C, R306C, and R294X) [27].

The case reports on fracture in patients with Rett syndrome are summarized in Table 2.

Three case reports describe the management of particular clinical situations in subjects with a history of fractures [29,30,34]. The remaining case reports describe the pharmacological management of the fragility fractures. In particular, the use of the teriparatide in subjects over the age of 18 years [32,33] and the use of bisphosphonates sequentially to the anabolic treatment [32] or alone [31]. These case reports show that any treatment used has proven effective in reducing the risk of fractures.

**Figure 2:**
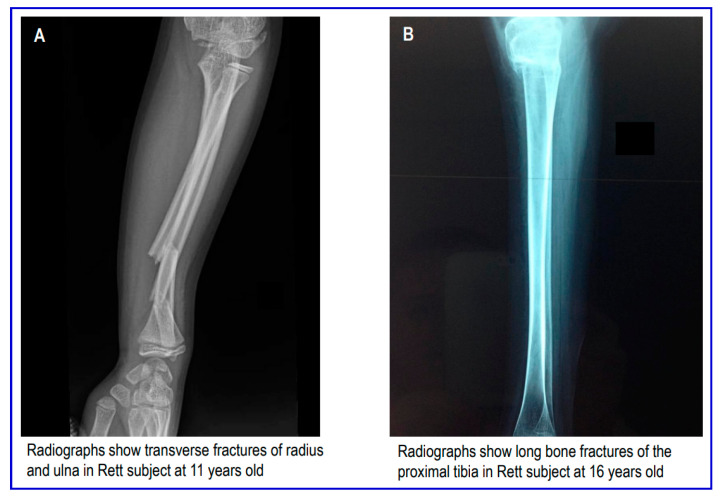
Types of fractures in Rett subjects at upper (**A**) and lower (**B**) extremities.

Moreover, in Figure 2, we show typical fractures that occurred in Rett subjects at upper and lower extremities.

## 4. Pathophysiology of Bone Fragility in Patients with Rett Syndrome

**Figure 3:**
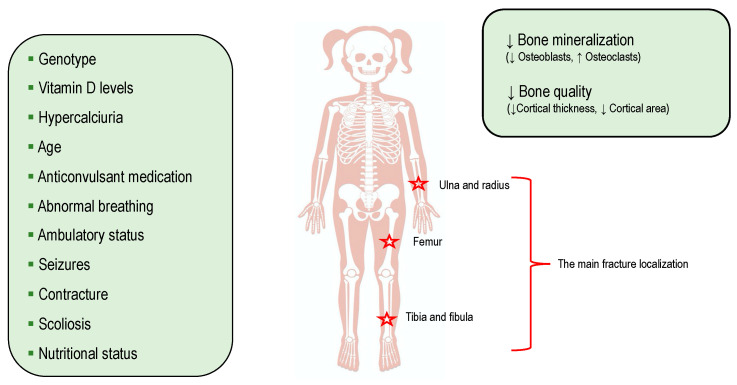
The main determinants and localization of fractures in Rett syndrome.

Several hormonal and environmental factors contribute to fragility fractures in patients with Rett syndrome. Bone is an extremely dynamic and plastic tissue that responds to stimuli mechanics through modifications of its own structure. Any kind of strength applied to the bone determines, proportionally to the strength itself, a bone deformation, transient or permanent. According to Frost’s mechanistic theory, the greater the deformation, the greater the periostal bone regeneration induced by mechanical stress [35]. The loss of the mechanical factor at a pediatric age can affect skeletal development and the achievement of an adequate bone mass peak [35]. The reduction in walking capacity or complete immobility leads to a reduction in bone mineral density, resulting in increased fractures for minor trauma [36]. Similarly, in a previous study by Presedo A et al. that identified a risk factor for fracture in a population of patients with cerebral palsy, the most often affected sites were the distal femur or proximal tibia [37]. Moreover, subjects with Rett syndrome are characterized by a slow rate of bone formation due to dysfunctional osteoblast activity rather than an increase in bone resorption [6]. Studies in humans and animals support the idea that the MECP2 mutation could be associated with altered epigenetic regulation, signaling pathways, and bone-related factors [6]. In particular, in two studies conducted in Mecp2-null mice and HET mice, the osteoblast morphology was altered and the osteoblast number was decreased. Therefore, when compared to WT mice, HET and Mecp2-null mice showed decreased rates of mineral apposition, a mineralizing surface, and a bone formation rate/bone surface [38,39]. Moreover, a study by Motil KJ et al. reported that the Rett syndrome cohort showed low osteocalcin levels, suggesting that the rate of bone formation was depressed, not only throughout childhood but especially during the adolescent growth spurt [40]. These data are also confirmed by Roende G et al., who found that girls with Rett syndrome seem to have a low bone turnover in the modeling period of childhood and youth [41]. Bone damage is frequently associated with nutritional deficiencies that are linked to difficulty in feeding, which can result in reduced bone formation and increased bone resorption. Moreover, the genotype is responsible for clinical pictures of severe bone disease with an increased risk of fracture. Epilepsy is a risk factor that affects almost all people with Rett syndrome. In fact, compared to the healthy general population, subjects with epilepsy have a doubled risk of vertebral fractures. Two physiopathological mechanisms justify this risk: the increased risk of falls during seizures and the effects of AED drugs on the bone [7,8]. Older-generation AEDs, such as phenytoin, phenobarbital and carbamazepine, exert a p450-inducing cytochrome function, reducing bone mineral density and interfering with vitamin D metabolism. Instead, new-generation AEDs have a lower impact on the function of cytochrome p450 and would therefore appear to be safer for the osteoskeletal well-being of patients [42]. The prevalence of vitamin D deficiency in Rett patients is high [15,20]. Certainly, among the risk factors that determine low serum vitamin D levels in these patients, it must be considered that most girls with Rett syndrome have limited outdoor sunlight exposure, a decreased dietary intake of vitamin D, and, above all, the use of medications such as anticonvulsants. Therefore, low serum vitamin D levels have been recognized as a risk factor for osteoporotic fractures in both children and adults [9]. The main fracture localization and the possible determinants of fractures in patients with Rett syndrome are shown in Figure 3.

## 5. Bone Health Prevention and Management Recommendations in Patients with Rett Syndrome and Fragility Fracture

**Figure 4:**
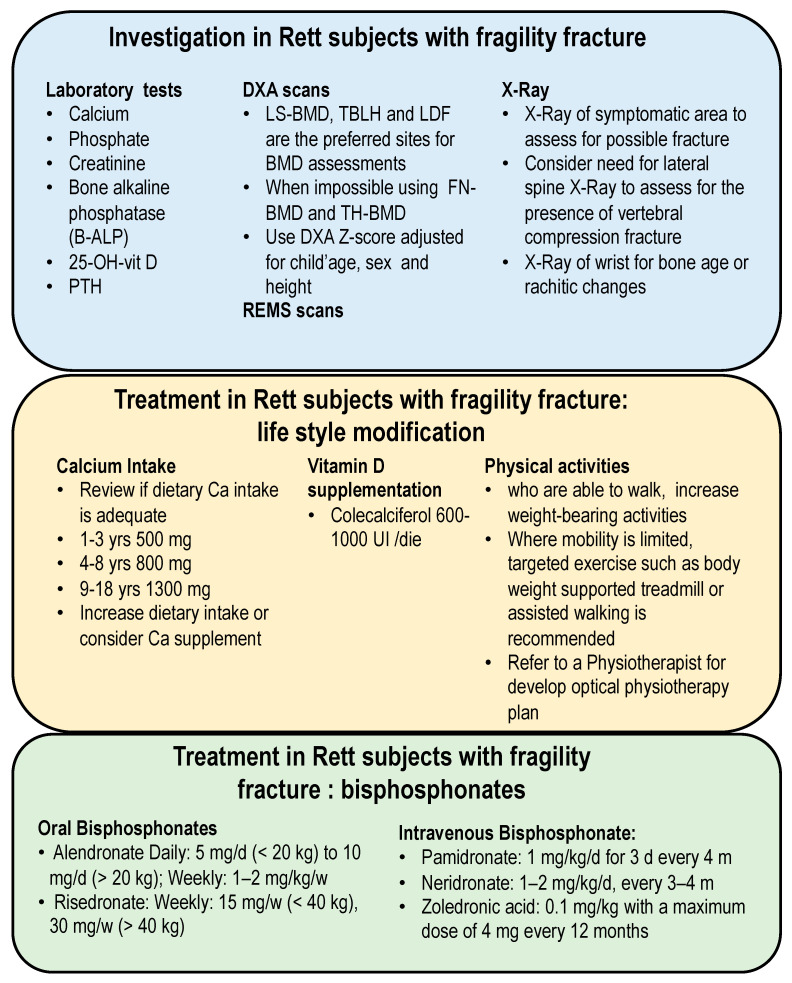
Suggested diagnostic and therapeutic care pathway for the management of Rett subjects with fragility fracture.

There is still no consensus on the treatment of fragility fractures in children. Controlled studies in children are lacking, and only a few have been conducted on sufficiently large samples. Especially for a rare disease such as Rett syndrome, there are no studies on the adequate treatment of low BMD that results in fractures. The previous consensus on the management of patients with Rett syndrome reported recommendations on the assessment and prevention of low BMD values without focusing on the treatment of fractures [43,44]. (Figure 4 reports a possible diagnostic and therapeutic care pathway for the management of patients with Rett syndrome and fragility fracture).

The first step for a correct diagnosis and management of bone disease in subjects with Rett syndrome is the biochemical assessment. In fact, for an appropriate diagnosis to rule out the secondary causes of osteoporosis, it is necessary to evaluate skeletal turnover. Bone mineral density (BMD) should be measured using dual-energy X-ray absorptiometry (DXA) in patients with Rett syndrome who are older than three years old and have a history of fragility fractures. According to the ISCD, the posteroanterior lumbar spine, the total body in children three years of age or older, and the lateral distal femur (LDF) measurements are the two preferred sites for pediatric DXA assessment. Finally, this site evaluates bone density at a weight-bearing, readily accessible, mechanically loadable skeletal site that is also a clinically relevant fracture location for patients with conditions that impair mobility, such as Rett syndrome subjects [45,46]. In children at risk for bone fragility who would benefit from the continuity of DXA measurements through the transition into adulthood, or in children with reduced weight bearing and mechanical loading of the lower extremities, proximal femur DXA measurements can be used, if reference data are available [45,46]. Low bone mineral mass or bone mineral density is defined for pediatric DXA reports when BMC or areal BMD Z-scores are less than or equal to −2.0 SD [45,46]. Plain radiographs are useful for identifying fractures and can be performed at any age. It is well known that patients with Rett syndrome have severe impairments in mobility status, which do not allow for the correct execution of a DXA scan. Alternatively, an assessment by quantitative ultrasound (QUS) or the Radiofrequency Echographic Multi Spectrometry (REMS) technique could be carried out directly in the bed of the patient and in the absence of ionizing radiation [47,48]. Alternatively, bone turnover markers (BTMs) could be evaluated, but to date, in children or adolescents, there is no evidence for the use of BTMs in clinical practice. These evaluations are still limited due to the many specificities related to children [49].

Concerning the management of fractures in patients with Rett syndrome, a rational strategy should be taken to recognize and, whenever possible, reduce or eliminate all risk factors that affect bone density and bone mass. Physical activity is crucial for boosting bone mineral density (BMD), not only in healthy individuals but also in those with various pathological conditions, such as Rett syndrome patients. In particular, as a general rule, therapeutic interventions must be prudent, beginning with the simplest and safest ones. Moreover, an adequate calcium intake must be ensured in relation to the specific needs of age. Furthermore, it is necessary to avoid a lack of vitamin D by intervening with adequate supplements [43,50]. Bisphosphonates (BPs) are the most used drugs so far in osteogenesis imperfecta and secondary pediatric osteoporosis and are indicated in children with a history of atraumatic fractures [51]. Bisphosphonates are, to date, the first-line therapy, and the most frequently administered are intravenous BPs such as pamidronate, neridronate, and zoledronate.

Intravenous pamidronate is undoubtedly the most widely used bisphosphonate, although there is no strong evidence regarding the dosage, duration of therapy, and, above all, long-term safety. The recommended doses for pamidronate range from 0.5 to 1 mg/kg/day, given for 3 consecutive days every 3 months. More recent data on bisphosphonates with a greater power of action have made it possible to formulate therapeutic schemes with less frequent administration, which are more pleasing to patients. Six-monthly intravenous infusions of neridronate (2 mg/kg) or zoledronate (0.025–0.05 mg/kg) have been shown to increase BMD and reduce the risk of fracture [51,52]. The decision to use or discontinue bisphosphonates during fracture healing should be made on a case-by-case basis, taking into account the individual’s overall health, the nature of the fracture, and the potential risks and benefits associated with bisphosphonate treatment.

Teriparatide significantly reduces, in adults, the risk of vertebral and non-vertebral fractures and appears to be an option of considerable interest for post-epiphyseal fusion children, particularly in the form of severe osteoporosis with multiple fragility fractures. Although teriparatide is not approved in pediatric patients and concerns remain regarding its use in subjects with unsealed epiphyses, preclinical studies on mouse models [53] and those on humans support the idea of studying its use in the treatment of osteoporosis associated with Rett’s syndrome, a disease in which vertebral and non-vertebral fractures are frequent to the point of compromising walking even permanently. In this regard, in the literature, there is only one case in which, after six months of therapy with teriparatide, there was a significant improvement in both densitometric and ultrasonographic values and no new fracture [32].

The surgical treatment of fractures in children is rarely indicated. First of all, a clear definition of the fracture is necessary to estimate the prognosis, establish an appropriate treatment plan, and comprehend the injury mechanism. Non-surgical treatments are often used to repair fractures in children due to their simpler healing process, higher remodeling ability, and low non-union rate. Children who have multiple injuries, open fractures, some pathologic fractures, fractures with coexisting vascular injuries, fractures with a history of unsuccessful conservative treatment, and fractures for which conservative treatment is ineffective or useless, such as fractures of the femur neck, some physeal fractures, displaced extension- and flexion-type humerus supracondylar fractures, displaced lateral condyle fractures in the humerus, and fractures in the femur and tibia, are all better treated surgically. In this case, the goal of treatment is not to interfere with the activity of the growth plate and to avoid any type of lesion of the growth cartilage. The most often used fixation methods are: percutaneous Kirschner threads, percutaneous cannulated screws, endomedullary elastic nailing according to the Métaizeau technique, and external fixation (exposed fractures). The use of endomedullary nails and screw plates is reserved for adolescents and must be evaluated on a case-by-case basis [54].

## 6. Conclusions

This manuscript, being based on studies carried out on small and heterogeneous populations, does not allow for drawing solid conclusions. However, the analysis of the data present in the literature permits us to obtain useful considerations for the management of subjects with Rett syndrome.

In particular, fractures are a common occurrence in patients with Rett syndrome. Around one-quarter of all patients suffer at least one fracture. Most of the fractures in patients with Rett syndrome are located in the upper extremities, in the lower extremities, and, in particular, at the femur. The site of a fracture often depends on the mechanism of the injury and the pathophysiology of the disease. Moreover, factors influencing the fracture risk in patients with Rett syndrome include the bone mineral content and density, calcium and vitamin D, and physical activity.

In the management of patients with Rett syndrome and underlying bone fragility, it is important to reduce the exposure to potential bone adverse factors, encourage weight-bearing physical activity, and maintain an appropriate nutritional intake, particularly of calcium and vitamin D supplementation. Bone density testing may be considered to assess bone health. Pharmacologic intervention may be warranted, depending on the severity of the bone phenotype, the underlying bone fragility condition, and the presence of bone pain. The most commonly used medication for treating bone fractures is bisphosphonates.

In addition, this work aims to highlight the problem of skeletal fragility in this category of patients. In fact, it is important to underline that the management of fragility fractures in individuals with Rett syndrome should be personalized, taking into account the individual’s unique needs, abilities, and overall health status. Consultation with a multidisciplinary healthcare team, including a pediatrician, orthopedic specialist, and physical therapist, is recommended for comprehensive care.

Further controlled multicenter studies are warranted in order to improve the treatment and management of bone fragility in patients with Rett syndrome.

## Figures and Tables

**Table 1:** The selected studies evaluating fractures in patients with Rett syndrome.

Study/Years/ Country	Subjects	Primary Measures	Bone Evaluation	Results	Conclusions
Loder RT (1989) USA [11]	36♀ (8.7 years)	n.a.	Radiographs	Fx = 10 in 5 pz (13.9%) 3 upper-extremity (humerus, clavicle, and wrist) 7 lower-extremity (4 femur, 2 tibia, and 1 toe)	Fx occured mainly at the upper extremities. Most of the Fx occurred during seizure. All RTT with TX had osteopenia.
Leonard H (1999) Australia [12]	101♀ (10.84 ± 4.52 years)	Metacarpophalangeal pattern (MCPP)	Hand radiographs	Fx = 35 (34.7%) 12 femur	The most common fx site was the femur. Fx were significantly more common in the group with moderate or severe OP. Bone quality was compromised (↓ cortical thickness and ↓ % cortical area).
Zysman L (2006) Israel [13]	35♀ (12.8 ± 8.8 years)	Tibia SOS Z-score = −1.05	QUS Omnisense 7000P	Fx = 7 (20%)	Fx correlate negatively with bone strength values as BMD.
Motil KJ (2008) USA [14]	50♀ (2–38 years)	WB-BMC Z-score = −2.2 ± 1.0 WB-BMD Z-score = −1.7 ± 1.2	DXA	Fx = 13 (28%)	Fx were associated with lower WB BMC and BMD.
Gonnelli S (2008) Italy [15]	Longitudinal 109♀ (10.1 ± 6.1 years)	AD-SoS Z-score = −2.01 ± 1.24 BTT Z-score = −1.89 ± 1.31	QUS Bone Profile-IGEA	Fx = 5 (5%) during 3 years of follow-up 2 at distal metaphysis, 1 at radial diaphysis, 1 at humerus, and 1 at peroneal diaphysis	All the fractures were consequences of minimal traumas. All but one had AD-SoS and BTT values lower than −2.0 Z-scores.
Down J (2008) Australia [7]	234♀ (14.7 years—range: 2–29 years)	n.a.	Survey Questionnaire	Fx = 84 (36%) [32 pz (32%) > 1 Fx] (head = 3.3%; trunk = 11.9%; upper limb = 30.5%; lower limb = 54.3%)	The femur and lower limb were the most common sites of fractures. Genotype p.R270X was more vulnerable to fractures. Epilepsy increased the fracture risk.
Leonard H (2010) Australia [16]	233♀ (14.7 years—range: 2–29 years)	n.a.	Survey Questionnaire	Fx = 84 (36.1%); 52 (22.3%) = 1 fx; 18 (7.7%) = 2 fx; 7 (3.0%) = 3 fx; 6 (2.6%) ≥ 4 fx.	After using valproate for a year or longer, the risk of fracture increased threefold.
Jefferson AL (2011) Australia [17]	97♀ (15 ± 7.1 years)	4–8 years: LS Z-score = −1.4; FN Z-score = −1.0 >8–14.5 years: LS Z-score = −1.21; FN Z-score = −1.63 > 14.5–20 years: LS Z-score = 0.46; FN Z-score = −1.31 > 20–31 years: LS Z-score = 1.11; FN Z-score = −3.07	DXA	Fx = 26 (31.7%)	BMD were particularly low at the FN, the most common site of fractures.
Roende G (2011) Denmark [8,18]	61♀ (20.1 years—range: 6.0–60.6 years)	aBMD-LS = 0.637 [0.389–1.183] vBMD-LS = 0.228 [0.145–0.335] aBMD-FN = 0.541 [0.302–0.840] vBMD-FN = 0.243 [0.118–0.414]	DXA	Fx = 14 (23%) (Humerus = 4; ulna and radius = 3; femur = 5; tibia, fubula, and patella = 3)	Low-energy falls in the lower limb occurred early in patients with RTT due to low-energy falls during regular daily activities [8]. Low bone mass, low BMD, and small bones seem to be associated with low-energy fx occurrence [18].
Motil KJ (2012) USA [19]	983♀ (8.7 years)	n.a	Survey Questionnaire	Fx = 294 (30%)	According to the current study, RTT children have a three- to fourfold higher fracture prevalence than do healthy children.
Sarajlija A (2013) Serbia [20]	35♀ (11.5 years; [IQR 6.8–17 years])	n.a	Medical history	Fx = 6 (17.1%) (3 femur, 3 radius, and 1 tibia)	Fx were induced by mild to moderate trauma. All the patients have been receiving AEDs at the time of the fracture. Carriers of R255X had a statistically higher fx risk. Vit D concentration was lower in RTT with fx.
Afzal SY (2014) USA [21]	14♀ (9.0 ± 4.4 years)	LS BMD Z-score = −2.66 ± 1.46 DF BMD Z-score = −4.20 ± 2.04	DXA	Fx = 5 (35.7%)	Conclusion: do not consider the presence of fractures in RTT.
Jefferson AL (2015) Australia [22]	Longitudinal 97♀ (15.2 years; 4.4–30.5 years)	LS BMD Z-score (−0.24 ± 1.5) WB BMD Z-score (−0.37 ± 1.3)	DXA	Fx = 5 (5%) during follow up (3–4 years) Fx = 35 (47.3%) during entire life	RTT sustained a fourfold greater incidence of Fx compared to an equivalently aged female population.
Horne T (2016) Australia [23]	255♀ (15.2 years; 4.4–30.5 years)	n.a.	Survey Questionnaire	Fx = 39 (15.3%) 18 femur	In RTT, difficulties in pain recognition increased the time needed to diagnose fx.
Lambert AS (2017) France [24]	All fractured 20♀ (12.5 years)	LS BMD Z-score (−3.2)	DXA	Fx = 37 (femur = 5 tibia = 6; humerus = 5; foot = 4; spine = 4; others = 13)	IV bisphosphonates constitute a beneficial adjuvant treatment for diminishing the risk of Fx and restoring BMD.
Wiedemann A (2018) France [25]	8♀ (12.3 years)	LS BMD Z-score = −2.97 FB BMD Z-score = −4.15	DXA	Fx = 2 (25%)	Annual ZA administration: (1) improves BMD, (2) improves quality of life, (3) decreases the incidence of fx, (4) decreases pain.
Caffarelli C (2019) Italy [26]	Longitudinal 58♀ 28 non ambulatory (5.1 ± 2.1 years) 30 ambulatory (5.9 ± 3.2 years)	BTT Z-score −1.91 ± 0.30; AD-SoS Z-score −2.27 ± 1.06	QUS Bone Profile-IGEA	Fx = 9 (15.5%) (Fx = 1 (3.3%) in ambulatory; Fx = 8 (28.6%) in non-ambulatory) Fx = 3 (5.2%) during 10 years of follow-up in non-ambulatory subjects only	Bone status in RTT appears to deteriorate due in part to ambulatory impairment and nutritional status.
Caffarelli C (2020) Italy [27]	125♀ (13.8 ± 8.3 years)	*More severe mutation* FNZ-score = −2.86; THZ-score = −3.44; WBZ-score = −2.13; AD-SoSZ-score = −2.29; BTTZ-score = −2.64 *Less severe mutation* FNZ-score = −1.34; THZ-score = −1.75; WBZ-score = −1.87; AD-SoSZ-score = −2.4; BTTZ-score = −2.51	DXA QUS QUS Bone Profile-IGEA	Fx = 19 (15.2%) *More severe mutation* Fx = 11 (17.1%) *Less severe mutation* Fx = 8 (12.0%)	Compared to the group with less severe mutations, Fx is more prevalent in the RTT group with more severe mutations. The history of fractures predicted WB-BMD.
Gripp EW (2021) USA [28]	11♀ (age 8.4 years)	LS BMD Z-score (−1.6) LDF BMD Z-score (−3.0)	DXA	Fx = 2 (18.2%) (femur = 2; finger = 1; humerus = 1)	The patients with a history of fracture had the lowest BMD.

Abbreviations: ♀: female; Fx: fracture; RTT: Rett syndrome; MCCP: metacarpophalangeal pattern; OP: osteoporosis; QUS: quantitative ultrasound: SOS: speed of sound; DXA: dual-energy X-ray absorptiometry; BMD: bone mineral density; WB: whole body; BMC: bone mineral content; BTT: bone transmission time; AD-SoS: amplitude-dependent speed of sound; LS: lumbar spine; FN: femoral neck; IQR: interquartile range; AEDs: antiepileptic drugs; RTT: Rett syndrome; IV: intravenous; ZA: zoledronate; TH: total hip; LDF: lateral distal femur.

**Table 2:** The case reports on fracture in patients with Rett syndrome.

Study/Years/Country	Study Characteristics	Primary Measures	Bone Evaluation	Results	Conclusions
Budden SS (2005) USA [29]	♂ (5 years)	BMD	DXA	Fx = left femur	A rare MECP2 mutations in a male.
Byard RW (2006) Australia [30]	♀ (20 years)	n.a.	n.a.	Fx = left tibia	Complication in Rett girls who died.
Lotan M (2013) Israel [31]	♀ (5 years)	Z-scores BMD Test I (age 5.5) −3.8 Test II (age 9) −1.3	DXA	Fx = 6 in 3 years	The ability to reverse the progression of osteoporosis and multiple fractures with bisphosphonates e.v.
Caffarelli C (2015) Italy [32]	♀ (18 years)	WB Z-score = −5.2 AD-SoS Z-score = −3.08 BTT Z-score = −3.30	DXA QUS Bone Profile IGEA	Fx = right tibia and fibula (3 years); left tibia and left capital ulnar (7 years); right humerus (16 years); right tibia (17 years)	The effectiveness of teriparatide and neridronate e.v. in the management of osteoporotic fractures in one subject with RTT.
Zanchetta MB (2016) Argentina [33]	♀ (28 years)	LS Z-score = −3.2 FN Z-score = −3.1	DXA	Fx = tibia	In a patient with RTT, anabolic therapy with teriparatide resulted in a significant improvement in the bone microarchitecture.
Som A (2016) India [34]	♀ (16 years)	n.a.	n.a.	Fx = left femur	Various complicating issues are needed for the successful perioperative management of these patients.

Abbreviations: ♂: male; ♀: female; BMD: bone mineral density; DXA: dual-energy X-ray absorptiometry; Fx: fracture; MPCP2: methyl-CpG binding protein 2; RTT: Rett syndrome; WB: whole body; BTT: bone transmission time; AD-SoS: amplitude-dependent speed of sound; QUS: quantitative ultrasound; SOS: speed of sound; LS: lumbar spine; FN: femoral neck.

## Data Availability

Not applicable.
